# Habitat-based CT radiomics enhances the ability to predict spread through air spaces in stage T1 invasive lung adenocarcinoma

**DOI:** 10.3389/fonc.2024.1436189

**Published:** 2024-10-11

**Authors:** Xiuhua Peng, Hongxing Zhao, Shiyong Wu, Dan Jia, Miaomiao Hu, Biping Guo, Jinliang Hu, Pengliang Xu

**Affiliations:** ^1^ Department of Radiology, The First People’s Hospital of Huzhou, Huzhou, China; ^2^ Department of Respiratory Medicine, The First People’s Hospital of Huzhou, Huzhou, China; ^3^ Department of Ultrasound, The First People’s Hospital of Huzhou, Huzhou, China; ^4^ Department of Thoracic Surgery, The First People’s Hospital of Huzhou, Huzhou, China

**Keywords:** invasive lung adenocarcinoma, radiomics, habitat imaging, tumor microenvironment, air space spread

## Abstract

**Introduction:**

Spread through air spaces (STAS) represents a novel invasive pattern in lung adenocarcinoma (LUAD) and is a risk factor for poor prognosis in stage T1 LUAD. This study aims to develop and validate a CT habitat imaging analysis model for predicting STAS in stage T1 invasive LUAD.

**Methods:**

We retrospectively analyzed 217 patients with preoperative stage T1 invasive LUAD (115 STAS-positive and 102 STAS-negative cases, including 151 in the train set and 66 in the test set). Semi-automatic segmentation was performed on the regions of interest (ROIs) in all CT images, with an automatic 3mm expansion around the tumor, considering the intratumoral and peritumoral 3mm area. This area was divided into three sub-regions via K-means clustering, and 1197 radiomic features were extracted from each sub-region and the overall combined region. After dimension reduction through the Mann-Whitney U test, Pearson correlation analysis, and least absolute shrinkage and selection operator(LASSO), the best features for each sub-region and overall were selected. Models were then built using the selected radiomic features through the Adaptive Boosting (AdaBoost) and Multilayer Perceptron (MLP) classifiers. Four different models were established based on different sub-regions and the overall features. The performance of these models was evaluated through receiver operating characteristic curves (AUC) under the DeLong test, calibration curves via the Hosmer-Lemeshow test, and decision curve analysis to assess the performance of these features.

**Results:**

In this study, we evaluated the predictive performance of AdaBoost and MLP classifiers on rad feature models across various subregions and the overall dataset. In the test set, the AdaBoost classifier achieved a maximum AUC of 0.871 in Habitat 3, whereas the MLP classifier demonstrated slightly superior performance with an AUC of 0.879. Both classifiers exhibited high efficiency in habitat 3, with the MLP algorithm showing enhanced model performance.

**Conclusions:**

CT habitat imaging analysis for the preoperative prediction of STAS in stage T1 invasive LUAD shows satisfactory diagnostic performance, with the habitat3 model exhibiting the highest efficacy, reflecting tumor heterogeneity.

## 1 Introduction

Lung adenocarcinoma (LUAD) is one of the malignancies with high incidence and mortality rates globally, and early-stage diagnosis and treatment significantly impact patient survival rates (1). In recent years, with the advancement of low-dose helical computed tomography and increased public health screening awareness, the detection rate of early-stage lung cancer has been on the rise; according to guidelines by the National Comprehensive Cancer Network(NCCN)in the United States, the current standard treatment for stage T1 LUAD is radical pulmonary resection, specifically lobectomy (2). Numerous domestic and international studies have also suggested that sublobar resection for early-stage lung nodules not only preserves lung function but also reduces the incidence of postoperative complications (3, 4). Despite surgical treatment significantly enhancing the cure rate and survival of patients with stage T1 lung adenocarcinoma, the recurrence rate in these patients still stands at 20-30% (5). Therefore, identifying patients with stage T1 LUAD at high risk of recurrence to provide precise surgical strategies has become a focal point of current clinical research.

Spread through air spaces (STAS) is a dissemination mode specific to lung cancer, involving cancer cells detaching from the primary site and spreading to surrounding areas via respiratory movements, reimplanting in the respiratory tract and alveolar walls, and further growing, thus facilitating lung cancer metastasis. This concept was first introduced in 2015 and was quickly recognized as an independent risk factor for lung adenocarcinoma recurrence and poor prognosis (6). Current studies have explored the relationship between STAS and postoperative prognosis of lung adenocarcinoma, where STAS-positive patients exhibit higher local and distant recurrence rates and shorter recurrence-free survival (RFS) (7–9). Some scholars have compared lobectomy and sublobar resection in stage T1 LUAD STAS-positive patients, finding significantly increased recurrence and metastasis rates in sublobar resections, suggesting that STAS positivity is an independent risk factor for stage T1 LUAD (10). Therefore, preoperative prediction of STAS status is crucial for choosing the surgical approach for stage T1 LUAD. If STAS can be diagnosed preoperatively or intraoperatively, it would guide the selection of clinical surgical treatment methods. Due to the limited inflation of lung tissues in intraoperative frozen sections, their ability to predict STAS status is limited. Currently, choosing a preoperative surgical approach for stage T1 LUAD remains a challenge, and accurate preoperative prediction of STAS is a hot research topic and an essential means to improve lung cancer survival rates, offering significant guidance for planning clinical surgical strategies.

Currently, radiomics technology has been extensively validated for predicting air space spread in LUAD but is limited to traditional radiomic analysis, i.e., of the tumor and surrounding areas (11–15). Each tumor is not just a homogenous entity but a mosaic of unique microenvironments, or habitats, composed of clusters of voxels with similar characteristics, consisting of tumor cells with identical genotypes and phenotypes. Habitat imaging distinctly segments the tumor into these sub-regions, which can reflect the spatial heterogeneity within the tumor to a certain extent, providing a new perspective for understanding and predicting the invasive behavior of tumors (16, 17). Habitat analysis has been applied to other types of tumors, such as breast cancer (18, 19), glioma (20, 21), cervical cancer (22, 23), liver cancer (24), colorectal cancer (25), recurrence of non-small cell lung cancer (26), pulmonary metastases (27), etc., but reports on its application in predicting STAS in lung adenocarcinoma are yet to be seen. The purpose of this study is to use habitat imaging to segment tumor sub-regions and incorporate peritumoral imaging to characterize spatial heterogeneity more accurately, aiming to predict the STAS status in stage T1 invasive LUAD more precisely, thereby providing a new scientific basis for preoperative assessment and treatment decision-making in stage T1 invasive LUAD.

## 2 Materials and methods

### 2.1 Patient selection and clinicopathological information

**Figure 1:**
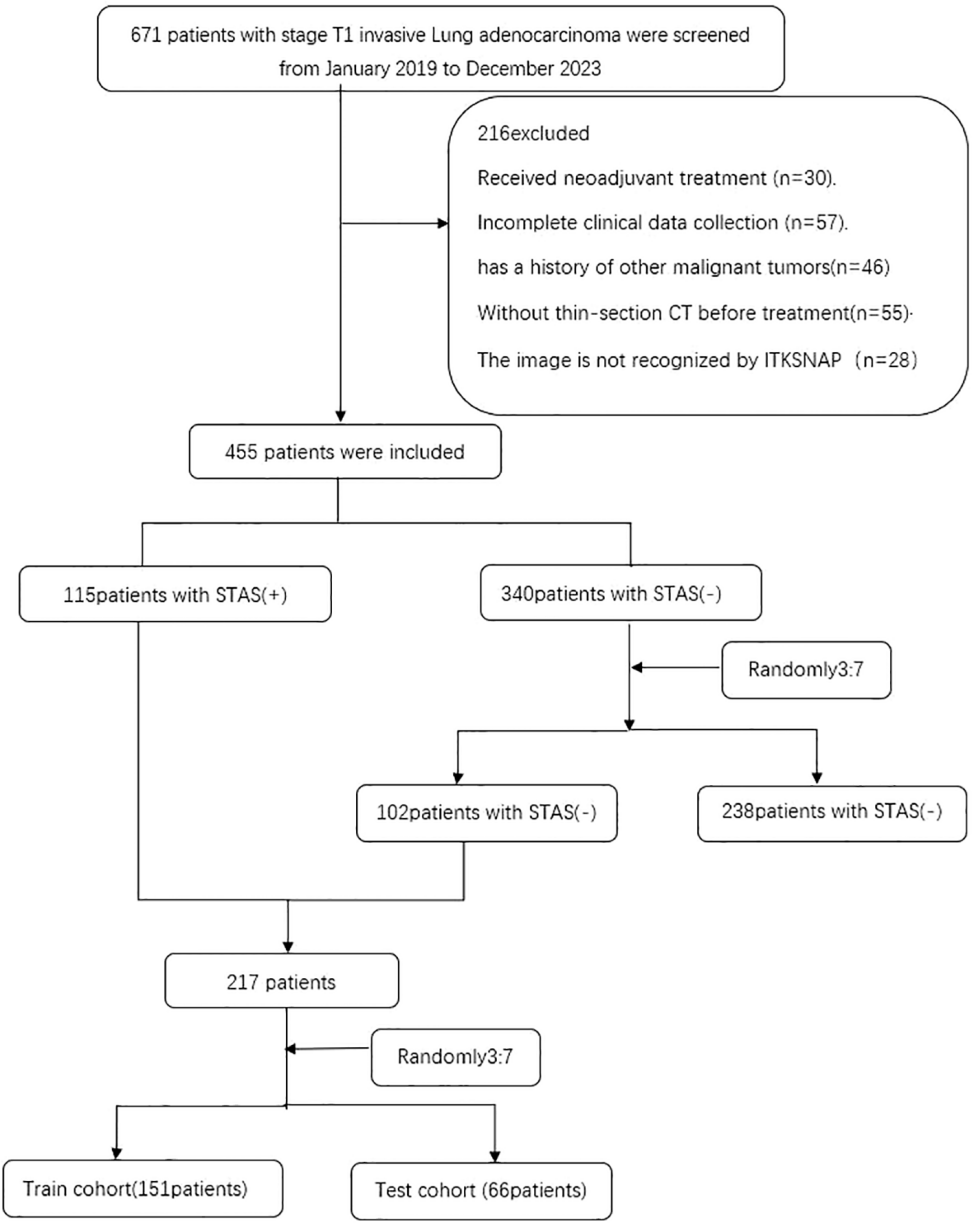
Flow diagram of the enrolment patients. STAS spread through air spaces; STAS(+), presence of STAS; STAS(-), absence of STAS.

This study follows the Declaration of Helsinki, has been approved by the Ethics Committee of Huzhou First People’s Hospital, and has waived the patients’ informed consent. The data of patients admitted to T1 invasive lung adenocarcinoma from January 2019 to December 2023. Inclusion criteria: (1) patients with maximum CT tumor diameter less than 3CM; (2) patients with CT imaging data within one month before surgery; (3) patients diagnosed with invasive lung adenocarcinoma; and (4) patients without distant metastasis before surgery. Exclusion criteria: (1) patients who had received neoadjuvant therapy; (2) patients with multiple pulmonary nodules reported on preoperative CT images; (3) patients with current or previous history of other malignancy; (4) patients with incomplete clinical data collection; and (5) patients whose images were not identified by ITK-SNAP. Ultimately, 455 eligible patients were continuously enrolled, of whom 115 were positive for STAS and 340 were negative for STAS. To overcome the possible imbalance in the data, we randomly grouped the STAS negative cases by 3:7 and matched them to an almost 1:1 ratio of the STAS positive group. This data balancing method has been demonstrated in previous studies (15), divided into a training group (n=151) and a validation group (n=66) (**Figure 1**).

Clinical and pathological variables included age, sex, serum tumor markers (carcinoembryonic antigen (CEA) and sugar antigen CA125 (CA125)), smoking status, tumor location, and emphysema; KI67, vascular infiltration, nerve infiltration, and invasion of pleural membrane.

### 2.2 Histopathological evaluation

According to the WHO, STAS is defined as the spread of micropapillary clusters, solid nests, or individual tumor cells into the alveolar spaces beyond the main tumor edge. The main morphological features include: (1) alveolar spaces filled with ring-shaped micropapillary structures without or with occasional central fibrovascular cores; (2) alveolar spaces filled with solid nests or tumor islands composed of tumor cells; and (3) alveolar spaces filled with discontinuous individual tumor cells. In this study, pathological examinations were reassessed and diagnosed by an attending physician with 5 years of experience and a chief physician with 15 years of experience, based on the WHO definition of STAS.

### 2.3 CT examination protocol

The chest scan was performed with German Siemens Definition AS 64-row 128-slice spiral CT. Scan from thoracic entrance to diaphragm level. The subjects were placed in the supine position and held their breath after deep inhalation. Scanning parameters: tube voltage 120kv, tube current 120mA, window width 1300-1500, window position: -600~-700, pitch 1.0, frame rotation time 0.33S/360 degrees. Lung window reconstruction was performed using the lung method with a reconstruction thickness of 1.25mm and layer spacing of 1.25mm. Mediastinal window reconstruction thickness and layer spacing were 5mm.

### 2.4 Image segmentation and data preprocessing

Image segmentation was performed independently by two radiologists with extensive experience. They were blinded to the patients’ histopathology. One of the radiologists (radiologist A, with 5 years of experience) manually drew the ROI slice by slice using the open-source software ITK-SNAP (version3.8.0, http://www.itksnap.org). Another radiologist (radiologist B, with 10 years of experience) reviewed all ROIs manually segmented by radiologist A. The software automatically expands its boundaries by 3mm to get a gross peritumor ROI. The 3mm area surrounding the tumor was manually removed where the soft tissue, bone, and mediastinum overlapped in the chest wall. Intra-class correlation coefficients (ICCs) are used to evaluate feature extraction. In terms of intra-observer and inter-observer consistency, ICCs≥0.75 indicates good consistency.

The dataset was randomly assigned in a 7:3 ratio to either the training dataset or the test dataset. All cases in the training dataset were used to train the predictive model, while cases in the test dataset were used to evaluate the model’s performance independently. Medical volumes are common with heterogeneous voxel spacing because of different scanners or acquisition protocols. Such spacing refers to the physical distance between two pixels in an image. Spatial normalization is often employed to reduce the effect of voxel spacing variation. The fixed-resolution resampling method was used in our experiment to handle the problems mentioned above. All images were resampled to a voxel size of 1*1*1 mm to standardize the voxel spacing. Finally, the data were standardized using z-score standardization (zero-mean normalization).

### 2.5 Sub-region clustering and feature extraction

Habitat utilizes voxel and entropy values from CT images to cluster VOIs into sub-regions (28–30). The voxel counts for each tumor VOI were determined using a traditional method, whereas the entropy values were computed for each layer of the CT images using the following formula:


$$V_{voxel}=\sum_{k=1}^{N_{V}}V_{k}$$

$$\text{ entropy }=\;-\sum_{\text{i}=1}^{{\text{N}}_{\text{g}}}\text{p}\left(\text{i}\right)\log_{2}\left(\text{p}\left(\text{i}\right)+\epsilon \right)\;=$$

The k-means method was employed to cluster the VOI regions at the patient level, forming multiple habitats, and the distance correlation between samples was calculated using the Euclidean distance (voxel values and entropy values). The number of habitats was tested from 2 to 10 to determine the optimized number of habitats with the highest evaluation metric, the Calinski–Harabasz index (31). The optimal k-value was the criterion for selecting the optimal number of clusters at the patient population level. The optimal k-value was found to be 3. Using Python software, we imported the volume of interest (VOI) for each patient into the system. The T1 aggressive lung adenocarcinomas were classified into three distinct categories: habitat 1, habitat 2, and habitat 3.

The handcrafted features can be divided into three groups: (I) geometry, (II) intensity, and (III) texture. The geometry features describe the three-dimensional shape characteristics of the tumor. The intensity features describe the first-order statistical distribution of the voxel intensities within the tumor. The texture features describe the patterns or the second and high-order spatial distributions of the intensities. Here the texture features are extracted using several different methods, including the gray-level co-occurrence matrix (GLCM), gray-level run length matrix (GLRLM), gray-level size zone matrix (GLSZM), and neighborhood gray-tone difference matrix (NGTDM) methods. Based on habitat imaging, image omics features were extracted from three subregions and the peri-tumor region, respectively. All features were extracted using the Pyradiomics package in Python version 3.9. Eight wavelet transform algorithms are used to obtain high-throughput features for first-order statistics and texture features, namely LLL, LLH, LHL, LHH, HLL, HLH, HHL, and HHH. Do z-score normalization for all features and change the feature value to 0 mean 1 variance.

### 2.6 Feature selection and model design

Statistics: We performed a Mann-Whitney U test and feature screening for all radiomic features. Only the radiomic features with a p-value< 0.05 were kept. Correlation: For features with high repeatability, Spearman’s rank correlation coefficient was also used to calculate the correlation between features, and one of the features with a correlation coefficient greater than 0.9 between any two features is retained. We use a greedy recursive deletion strategy for feature filtering to maintain the ability to depict features to the greatest extent. That is, the feature with the greatest redundancy in the current set is deleted each time. Lasso: LASSO regression model was used on the discovery data set for signature construction. Depending on the regulation weight *λ*, LASSO shrinks all regression coefficients toward zero and sets the coefficients of many irrelevant features exactly to zero. To find an optimal *λ*, 10-fold cross-validation with minimum criteria was employed, where the final value of *λ* yielded minimum cross-validation error. The retained features with nonzero coefficients were used for regression model fitting and combined into a radiomics signature. Subsequently, we obtained a radiomics score for each patient by a linear combination of retained features weighed by their model coefficients. The Python scikit-learn package was used for LASSO regression modeling.

After Lasso feature screening, we input the final features into the AdaBoost and MLP classifiers for risk model construction. Here, we adopt 5 5-fold cross-verification to obtain the final Rad Signature. Receiver operating characteristic (ROC) curves were plotted to assess the diagnostic performance of the predictive models, and the corresponding area under the curve (AUC), diagnostic accuracy, sensitivity, specificity, positive predictive value (PPV), and negative predictive value (NPV) were analyzed.

### 2.7 Statistical analysis

The Python state models (version 0.13.2) package was used to perform statistical analysis, and a p-value< 0.05 was considered statistically significant. We analyzed the differences between groups using Student’s t-test or Mann−Whitney U tests for continuous variables; the chi-square test or Fisher’s exact test was applied for categorical variables.

## 3 Results

### 3.1 Patient characteristics

A total of 217 patients, including STAS positive 115and STAS negative 102patients, were included in our study. Patients were divided into a training set (151 patients) and an independent test set (66 patients) based on treatment duration. A pathologist reviewed the pathological data. All patients underwent surgical treatment; there were 77 (51%) patients with STAS positive and 74 (49%) patients with STAS negative in the training group and37 (56%) patients with STAS positive and 29(44%) patients with STAS negative in the test group.

The characteristics of the patients in the cohort are shown in **Table 1**. The comparison of age, gender, CA125, smoking status, lobular locations, Vascular infiltration, perineural invasion, and Pleural infiltration showed no significant difference between the two groups and within each group (p>0.05), ensuring a reasonable classification. Significant differences between the cohorts were found in CEA (p<0.05).

**Table 1:** Baseline characteristics of patients in the training cohort and test cohort.

Characteristics	Total (N=217)	Train(N=151)	Test(N=66)	P-value
Age (years)	64.96 ± 9.58	65.44 ± 9.21	63.88 ± 10.37	0.424
Maximum-diametere(cm) ()()(cm)	1.68 ± 0.64	1.67 ± 0.65	1.70 ± 0.61	0.574
Gender				0.07
Male	104(47.93)	79(52.32)	25(37.88)	
Female	113(52.07)	72(47.68)	41(62.12)	
Smoke				0.414
Non-smoker	164(75.58)	117(77.48)	47(71.21)	
Smoker	53(24.42)	34(22.52)	19(28.79)	
CEA				0.007
Negative	171(78.80)	111(73.51)	60(90.91)	
Positive	46(21.20)	40(26.49)	6(9.09)	
CA125				0.056
Negative	206(94.93)	140(92.72)	66(100.00)	
Positive	11(5.07)	11(7.28)	0	
Lobular locations				0.533
RUL	67(30.88)	48(31.79)	19(28.79)	
RML	27(12.44)	15(9.93)	12(18.18)	
RLL	31(14.29)	21(13.91)	10(15.15)	
LUL	59(27.19)	43(28.48)	16(24.24)	
LLL	33(15.21)	24(15.89)	9(13.64)	
Vascular_infiltration				1.0
No	142(65.44)	99(65.56)	43(65.15)	
Yes	75(34.56)	52(34.44)	23(34.85)	
perineural_invasion				0.984
No	212(97.70)	147(97.35)	65(98.48)	
Yes	5(2.30)	4(2.65)	1(1.52)	
Pleural_infiltration				0.235
No	179(82.49)	121(80.13)	58(87.88)	
Yes	38(17.51)	30(19.87)	8(12.12)	
KI67				0.827
<20%	181(83.41)	127(84.11)	54(81.82)	
≥20%	36(16.59)	24(15.89)	12(18.18)	
lymphatic_metastasis				0.707
No	193(88.94)	133(88.08)	60(90.91)	
Yes	24(11.06)	18(11.92)	6(9.09)	
Emphysema				0.214
No	143(65.90)	104(68.87)	39(59.09)	
Yes	74(34.10)	47(31.13)	27(40.91)	

LLL, left lower lobe; LUL, left upper lobe; RLL, right lower lobe; RML, right middle lobe; RUL, right upper lobe.

### 3.2 Workflow of radiomics analysis

**Figure 2:**
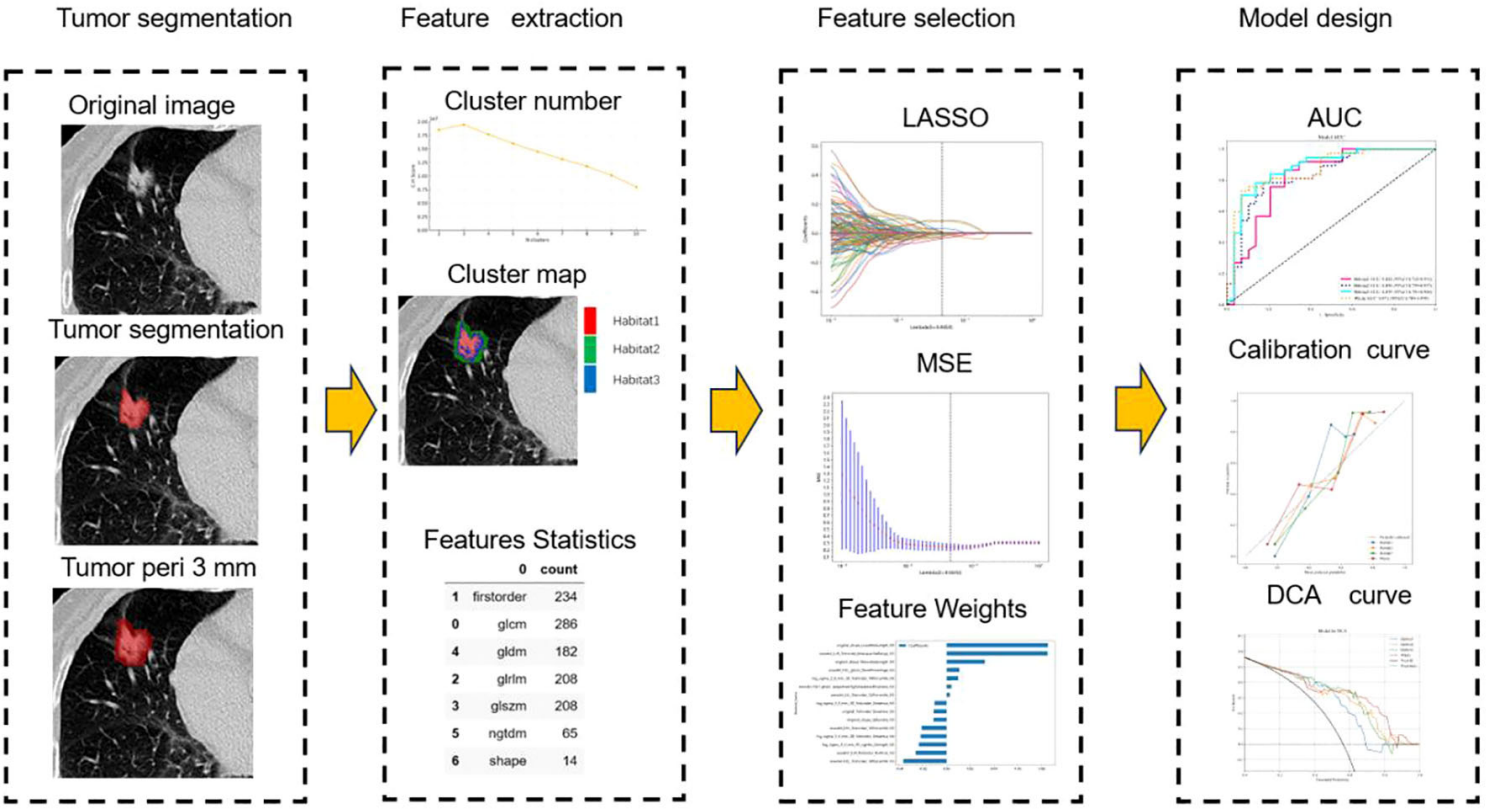
Workflow of radiomics analysis.

The radiomics analysis consisted of a series of steps: image segmentation, feature extraction, feature selection, signature construction, and evaluation (**Figure 2**).

### 3.3 Feature selection and radiomics signature development

**Figure 3:**
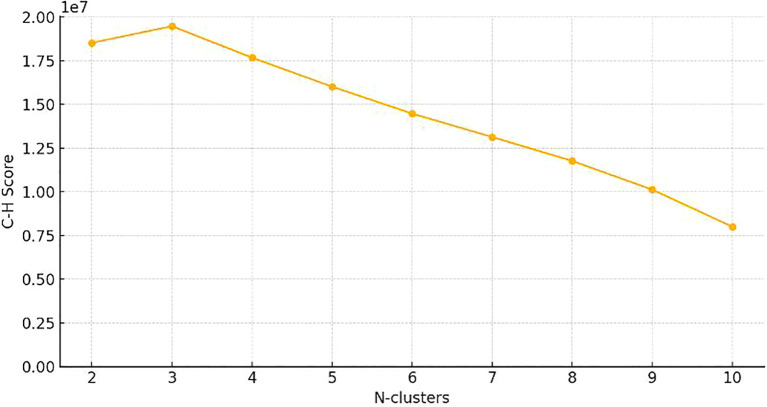
Calinski–Harabasz score plot. The red dotted line represented the optimal value beyond which the scores started to decrease in the radiomics features from CT images.

Features Statistics: Optimal CH value emerged when tumors were clustered into three sub-regions in the entire cohort (**Figure 3**). A total of 1197 manual features were extracted for each sub-region and population, among which 234 were the first feature, 14 were shape features, and the last were texture features. All handcrafted features are extracted with an in-house feature analysis program implemented in Pyradiomic (http://pyradiomics.readthedocs.io).

Feature selection: Through the Mann-Whitney U test, Pearson correlation analysis, and LASSO dimension reduction processing, we screened out the best features of each sub-region and the whole. Fifteen features were obtained in habitat 1. 23 features were obtained in habitat 2. Fifteen features were obtained in habitat 3. In the whole region, 20 features are obtained.

**Figure 4:**
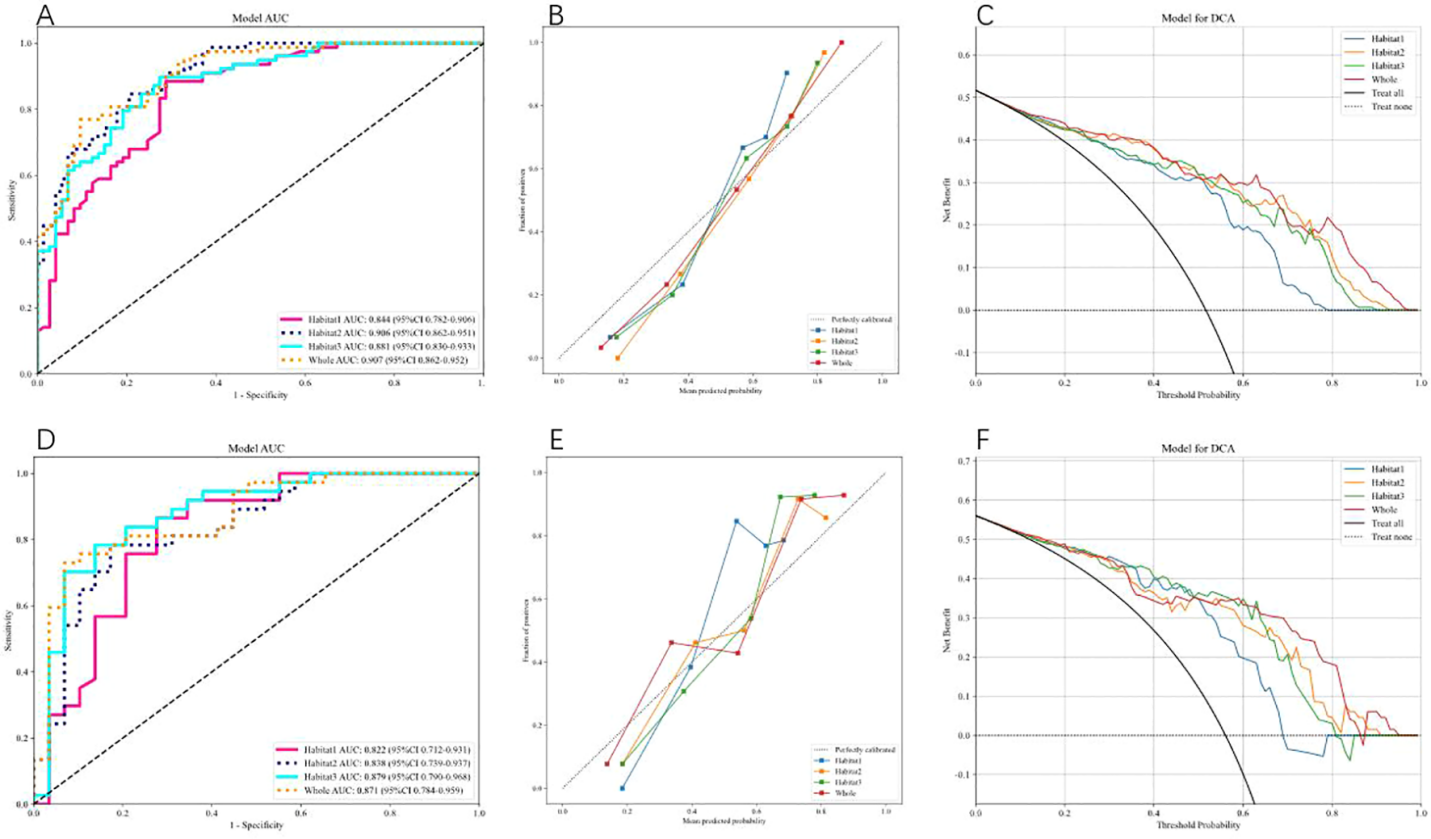
shows the Receiver Operating Characteristic (ROC) curve, correction curve, and decision curve analysis (DCA) for all features in the training cohort **(A–C)** and test cohort **(D–F)** of the MLP model.

**Figure 5:**
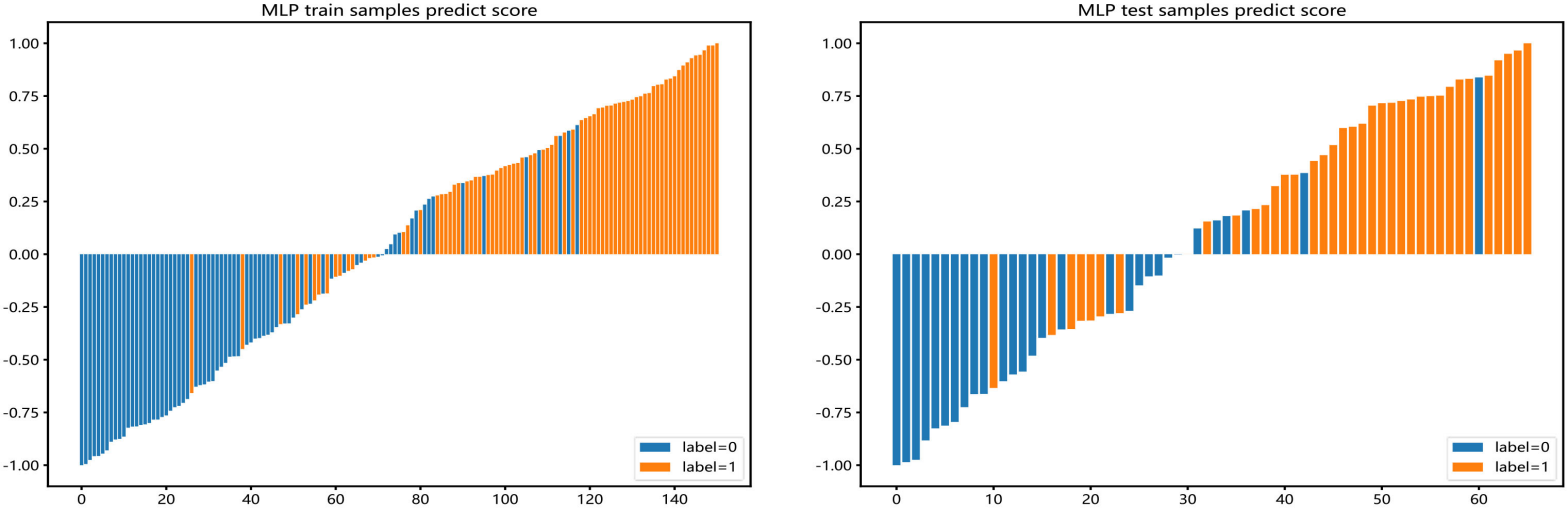
Barplots depicting the classification performance of radiomic features of habitat 3 in the MLP model. The yellow bar with a prediction value > 0 indicates that the signature successfully classifies the STAS patients; the red bar with a prediction value< 0 indicates that the signature fails to classify the STAS patients. For the blue bar, the contrary applies.

**Figure 6:**
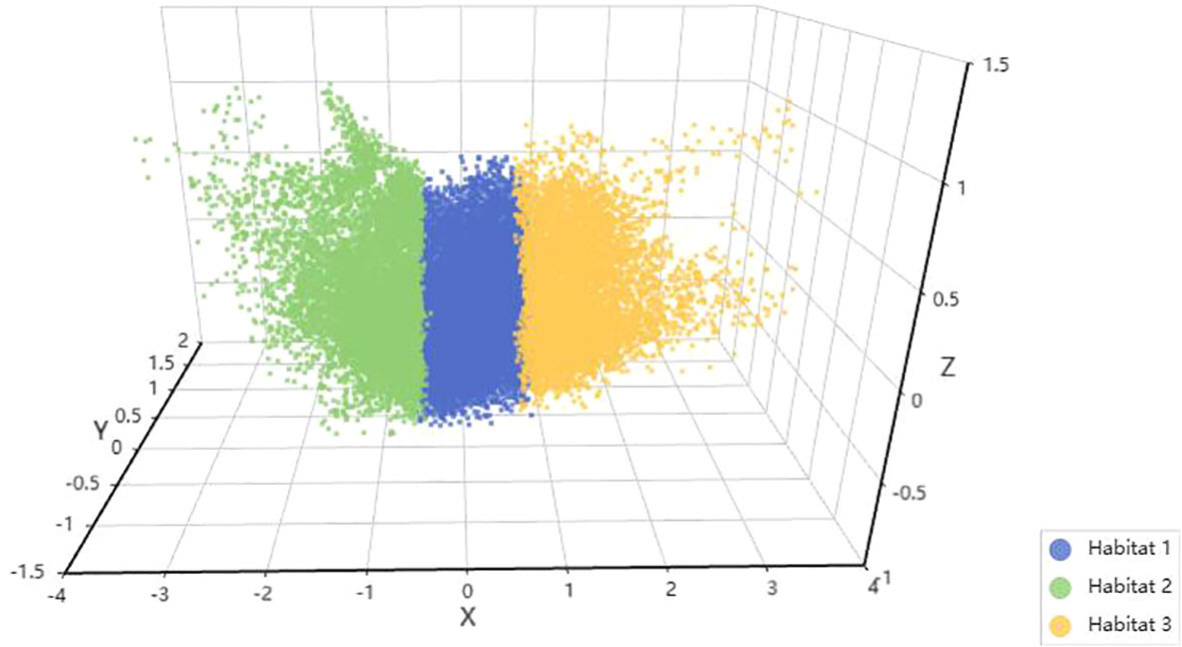
Habitat 3D visualization. Each habitat is represented by a different color: green for habitat 1, blue for habitat 2, and yellow for habitat 3.

We use AdaBoost and MLP classifiers, respectively, to predict rad feature models for each habitat. Among them, the test group habitat 3 of the AdaBoost classifier has the highest AUC, 0.871. The test group of the MLP classifier also has the highest AUC, which is 0.879. Therefore, among the two classifiers, habitat 3 has higher efficiency, and the model efficiency of the MLP classifier is higher. (**Tables 2**, **3**). **Figure 4** shows the receiver operating characteristic (ROC) curves, calibration curves, and decision curve analysis (DCA) for all features in the training and testing sets of the MLP model. Barplots depicting the classification performance of the habitat3 signature in the training and validation cohorts are shown in **Figure 5**. The distribution of three different habitats in a three-dimensional space is shown in **Figure 6**.

**Table 2:** Performance of each sub-region and the whole imaging rad model of the AdaBoost classifier in predicting STAS.

Signature	Accuracy	AUC	95% CI	Sensitivity	Specificity	PPV	NPV	Cohort
Habitat1	0.815	0.905	0.8608 - 0.9499	0.885	0.740	0.784	0.857	Train
Habitat2	0.881	0.957	0.9307 - 0.9838	0.885	0.877	0.885	0.877	Train
Habitat3	0.861	0.952	0.9230 - 0.9801	0.859	0.863	0.870	0.851	Train
Whole	0.854	0.940	0.9057 - 0.9745	0.795	0.918	0.912	0.807	Train
Habitat1	0.773	0.852	0.7553 - 0.9484	0.730	0.828	0.844	0.706	Test
Habitat2	0.727	0.802	0.6975 - 0.9064	0.757	0.690	0.757	0.690	Test
Habitat3	0.788	0.871	0.7845 - 0.9583	0.784	0.793	0.829	0.742	Test
Whole	0.727	0.816	0.7061 - 0.9258	0.676	0.793	0.806	0.657	Test

**Table 3:** Performance of each subregion and the whole imaging rad model of the MLP classifier in predicting STAS.

Signature	Accuracy	AUC	95% CI	Sensitivity	Specificity	PPV	NPV	Cohort
Habitat1	0.795	0.844	0.7824 - 0.9057	0.872	0.712	0.764	0.839	Train
Habitat2	0.815	0.906	0.8615 - 0.9509	0.833	0.795	0.812	0.817	Train
Habitat3	0.808	0.881	0.8295 - 0.9334	0.885	0.726	0.775	0.855	Train
Whole	0.828	0.907	0.8616 - 0.9516	0.756	0.904	0.894	0.776	Train
Habitat1	0.788	0.822	0.7119 - 0.9311	0.838	0.724	0.795	0.778	Test
Habitat2	0.788	0.838	0.7386 - 0.9371	0.757	0.828	0.848	0.727	Test
Habitat3	0.803	0.879	0.7900 - 0.9677	0.757	0.862	0.875	0.735	Test
Whole	0.803	0.871	0.7838 - 0.9590	0.703	0.931	0.929	0.711	Test

**Figure 7:**
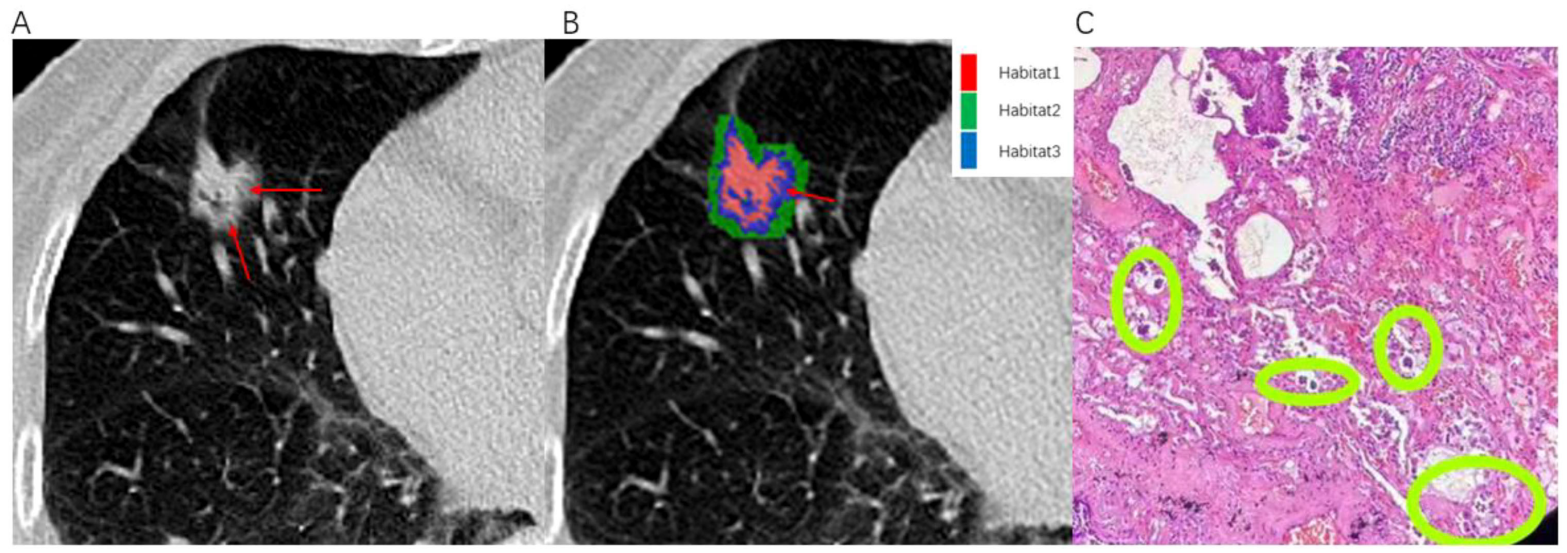
Illustrates an example of a patient with infiltrative pulmonary adenocarcinoma. An 80-year-old female presented with positive STAS associated with lung adenocarcinoma. **(A)** The axial CT image (width, 1300 HU; level, -300 HU) shows a mixed nodule in the right middle lobe, with ground-glass components indicated by the red arrow. **(B)** The habitat analysis image divides the lung nodule into three habitats, with the blue area representing Habitat 3 (indicated by the red arrow). **(C)** Microscopic photograph (Hematoxylin-Eosin staining, magnification 40x) reveals clusters of isolated small papillary tumor cells (within the green circle) present in the alveoli beyond the margins of the primary tumor.

CT images and histopathological photographs of STAS-positive nodule is shown in **Figure 7**.

## 4 Discussion

In this study, we utilized CT images to extract radiomic features from the tumor and an additional 3mm peritumoral sub-regions, establishing a multi-regional radiomics model for predicting the status of STAS. Results indicated that Habitat3 models exhibited robust predictive performance, with AUC values of 0.881 in the training set and 0.879 in the testing set using the MLP classifier. This approach, employing radiomics from the tumor and 3mm peritumoral sub-regions divided into different areas, is a novel method proposed for predicting STAS status, providing a robust basis for preoperative precise surgical planning for patients with stage T1 invasive LUAD.

Numerous researchers have explored radiomics to predict LUAD STAS. Chen et al. (32) segmented tumors in a cohort of 233 stage I LUAD patients, extracted radiomic features, and constructed a model to predict the presence of STAS. The model demonstrated AUC values of 0.63 and 0.69 during internal and external validation, respectively, underscoring CT-based radiomics’s utility in the preoperative STAS prediction in stage I LUAD. Han et al. (15) employed a similar radiomic approach on preoperative stage IA LUAD patients, achieving AUC values of 0.812 and 0.850 in the training and testing sets, respectively, using logistic regression. Jiang et al. (11) utilized the same segmentation technique on a dataset of 462 LUAD patients and developed a model using the random forest classifier, which achieved an AUC of 0.754. These studies focused solely on the tumor without analyzing the peritumoral area’s impact on STAS. Zhuo and Qi et al. (12, 33)studied various regions surrounding lung adenocarcinoma tumors and determined that an integrated model incorporating peritumoral areas along with other clinical parameters demonstrates higher efficacy than a radiomic model based solely on the tumor itself. These studies highlight that STAS occurrence is mainly related to the intrinsic aggressiveness of the tumor, with the peritumoral area also reflecting the tumor’s invasive behavior to some extent.

Our study combined features from the tumor and its surroundings, avoiding the oversight of additional value from the tumor microenvironment. We discovered that representing the spatial heterogeneity within different habitats of the tumor and its surroundings can better predict the status of STAS. Tumors themselves, as complex ecosystems akin to natural habitats, consist of heterogeneous sub regions that follow the principle of survival of the fittest, growing and increasing under various pressures, closely related to the progression and prognosis of the tumor. Based on radiomics principles and differences in pathology and biology, Habitat imaging uses quantitative imaging markers to comprehensively and non-invasively characterize the tumor environment, visualizing and quantifying the tumor’s internal heterogeneity. We obtained 1197 high-dimensional features from each sub region for STAS based on CT images. Therefore, feature selection is a critical step before constructing a radiomics model. Our study employed a three-step method for feature selection from different aspects. To describe tumor heterogeneity, we identified the best features from each sub region and the overall combined regions. We obtained 15 features in habitat1, 23 in habitat2, and 15 in habitat3, with 20 features in the whole area. The obtained features primarily focused on filtered image intensity and texture characteristics, exploring deeper correlations with tumor biological changes through filtering transformations.

We used the AdaBoost and MLP classifiers to predict rad feature models for each habitat and the whole. Among these, the test set for habitat3 had the highest AUC with the AdaBoost classifier (0.871) and the MLP classifier (0.879). Thus, habitat3 demonstrated high efficacy with both classifiers, with the MLP classifier showing higher model performance. AdaBoost’s main advantage lies in its ability to enhance difficult-to-classify samples, improving the overall accuracy of the model, and compared to some complex classifiers, it is more efficient in implementation and operation, making it a powerful and practical tool for various classification challenges. MLP, a type of feedforward artificial neural network, consists of multiple layers, including input, one or more hidden layers, and an output layer, with each layer composed of numerous neurons interconnected by learnable weights. Its main advantages are its strong non-linear learning capabilities and complex pattern representation ability, making it highly effective in many modern AI applications such as image recognition, speech processing, and complex classification tasks. The results obtained using these two classifiers established a more robust and resilient model.

This study concludes that the model constructed by habitat3 exhibits the highest efficacy, surpassing that of the whole model, indicating that intra-tumoral subregions are more indicative of STAS status than the tumor as a whole. This also suggests that habitat analysis reflects tumor heterogeneity more than traditional radiomics (22). Habitat3 represents the peripheral ground-glass opacity (GGO) components of the tumor, possibly due to tumor cells in the GGO area enhancing their migration and invasion capabilities by altering cell adhesion mechanisms and signaling pathways, leading to the occurrence of STAS (34). The GGO area typically reflects changes in the tumor microenvironment, including inflammatory responses and extracellular matrix remodeling, which may also promote the occurrence of STAS (35, 36). Habitat3 manifests radiologically as the range inward or outward from the tumor edge, corresponding to the radiological tumor margin, which aligns with the pathological tumor margin. It is also possible that tumor growth gradually expands outward, with central tumor cells gradually hypoxic and necrotic due to aggregation, resulting in the richest blood supply at the tumor’s edge, thus reflecting tumor heterogeneity most prominently. However, habitat2, representing the area surrounding the tumor, exhibits lower model efficacy than Habitat3, possibly due to interference from normal lung tissue in the peritumoral region. Previous studies (33) extracting peritumoral areas at 5mm, 10mm, and 15mm have yielded good predictive model performance but with deviations from the ideal curve in calibration. This suggests that predictive models based on radiomic parameters within lung tumors outperform those based on radiomic parameters from the peritumoral region, possibly due to greater interference from normal lung tissue surrounding the tumor. Some studies separately extracted radiomic features from the tumor and peritumoral regions at 3mm, 6mm, and 9mm, constructed models, and found that radiomic features from the tumor and peritumoral regions at 3mm can enhance the impact on the overall survival rate of patients with non-small cell lung cancer after surgery, and radiomic features from the peritumoral region at 3mm are associated with STAS (37). This study only extracted 3mm around the tumor for this reason. Previous methods of automatic expansion of peritumoral areas have been rigid and prone to interference, unable to reflect the true characteristics of the tumor surroundings accurately. This study utilizes unsupervised K-means clustering to categorize identical components within the tumor and peritumoral regions into the same subregion, resulting in a higher efficacy in predicting STAS status. Research indicates (38) that a 5mm expansion or contraction within the tumor edge yields higher predictive efficacy for STAS, similar to the results of this study, although with a lower AUC of 0.79 compared to our findings. This may be due to the automatic expansion of the previous study’s contraction area, whereas our study is based on clustering of identical components, resulting in slightly more precise results.

This study has several limitations: firstly, as a retrospective study with a small sample size from a single center, there may be potential bias. It is recommended that future studies be multi-centered to increase the number of cases and validate the model across different patient populations, thereby enhancing its generalizability and practicality. Secondly, this study utilized manual segmentation for ROI delineation, which may introduce subjective variability. Future research should employ automated segmentation techniques to mitigate these limitations and enhance segmentation consistency and accuracy. Additionally, this study only used conventional CT scans for subregion segmentation, not accurately representing the tumor’s information. Energy spectrum CT enhancement, containing more material energy and blood supply information, will be included in future research. Besides machine learning, deep learning is also becoming increasingly popular, and this aspect can also be explored in LUAD STAS habitat analysis.

In conclusion, this study constructed a CT habitat subregion radiomics model that non-invasively predicts the STAS status of T1-stage invasive lung adenocarcinoma preoperatively. The model has improved the robustness of predictive performance, providing a basis for distinguishing the benign or malignant nature of lung nodules and has auxiliary diagnostic value in assessing malignancy severity. The results offer a quantitative reference for preoperative surgical planning and postoperative chemotherapy selection in patients with T1-stage invasive lung adenocarcinoma, suggesting its potential as a non-invasive biomarker for preoperative STAS in lung adenocarcinoma patients.

## Data Availability

The original contributions presented in the study are included in the article/**Supplementary Material**. Further inquiries can be directed to the corresponding author.
